# Disparities in Hand Surgery Exist in Unexpected Populations

**DOI:** 10.7759/cureus.39736

**Published:** 2023-05-30

**Authors:** Laura A Stock, Jane C Brennan, Andrea H Johnson, Jeffrey Gelfand, Justin J Turcotte, Christopher Jones

**Affiliations:** 1 Orthopedic Research, Luminis Health Anne Arundel Medical Center, Annapolis, USA; 2 Orthopedic Surgery, Luminis Health Anne Arundel Medical Center, Annapolis, USA

**Keywords:** social vulnerability index, quick dash score, racial disparity, health care disparity, trigger finger release, carpal tunnel release

## Abstract

Background

The purpose of our study is to investigate disparities in the patient populations and outcomes of carpal tunnel release (CTR) and trigger finger release (TFR).

Methods

A retrospective review of 777 CTR and 395 TFR patients from May 2021 to August 2022 was completed. The shortened form of the Disabilities of the Arm, Shoulder, and Hand (DASH) scores (QuickDASH) was recorded to evaluate physical function preoperatively and at one and three months postoperatively. This study was deemed institutional review board-exempt by the institutional clinical research committee.

Results

Compared to CTR, TFR patients resided in zip codes with higher levels of social vulnerability across dimensions of ‘household composition and disability’ (p=0.018) and ‘minority status and language’ (p=0.043). When analyzing QuickDASH scores by demographics and procedure, preoperative scores were statistically significantly higher for non-married (p=0.002), White (p=0.003), and female sex (p=0.001) CTR patients. Further, one-month postoperative scores were statistically higher for White and non-married CTR patients (0.016 and 0.015, respectively). At three months postoperatively, female and non-married patients had statistically significant higher scores (0.010 and 0.037, respectively). In TFR patients, one-month postoperative QuickDASH scores for White and female patients were statistically significantly higher (0.018 and 0.007, respectively). There were no significant differences in QuickDASH scores between rural and non-rural patients, household income (HHI) above or below the median, or the Social Vulnerability Index (SVI) dimensions.

Conclusion

Our study found marital status, sex, and race were associated with disparities in pre-and postoperative physical function in patients undergoing carpal tunnel or trigger finger release. However, future studies are warranted to confirm and develop solutions to disparities within this population.

## Introduction

Carpal tunnel syndrome (CTS) and trigger finger (TF) are two of the most common nontraumatic hand disorders treated [1]. CTS has been reported to occur in 3%-5% of the general population and TF in about 2%-3% of the general population [2]. Both CTS and TF can cause significant disability and affect physical, mental, and social health [3]. Patients with worse mental health and higher levels of social deprivation also have an increased risk of poorer outcomes after surgical intervention for these conditions [3, 4]. Social deprivation has also been associated with worse patient-reported outcome measures (PROMs) in other specialties [5]. However, studies have shown over 90% patient satisfaction following surgical interventions for upper extremity diagnoses [6].

Disparities in healthcare utilization and outcomes are known to exist throughout the healthcare system and are an increasing focus of research and intervention. There are multiple ways to categorize and evaluate disparities to determine where interventions are needed. The Social Vulnerability Index (SVI) was developed by the Centers for Disease Control (CDC) to identify communities that are most likely to need support during hazardous events by looking at four themes, including socioeconomic status, household characteristics, racial and ethnic minority status, housing type, and transportation [7]. This has been defined for each census tract in the United States. Patients living in communities with higher SVI scores have been shown to have poorer health outcomes, particularly with regard to surgical outcomes [8-10].

Previous studies have investigated social and demographic factors to predict the likelihood of surgery; however, few have compared these factors by procedure type. The purpose of our study is to investigate disparities in patient populations and outcomes between patients undergoing carpal tunnel release (CTR) and trigger finger release (TFR).

This article has been accepted as a poster presentation at the American College of Surgeons Quality and Safety Conference, which will be held between July 10 and 13, 2023.

## Materials and methods

This study was deemed institutional review board-exempt by the institutional clinical research committee of Anne Arundel Medical Center, Annapolis, Maryland, USA. A retrospective chart review of all patients undergoing TFR or CTR at a single institution was performed. The timeline for inclusion was between May 2021 and August 2022.

A total of 1,172 patients underwent TFR or CTR over the study period and were analyzed. Patients were classified by whether they had a CTR (n=777) or TFR (n=395). Both open and endoscopic procedures were included in this study. To be included in the study, patients must have completed patient-reported outcome instruments at a minimum of one of the time points assessed.

Independent variables of interest included age, sex, race, marital status, household income (HHI), and the social vulnerability index (SVI), as shown by the four themes: socioeconomic status (SVI 1), household characteristics (SVI 2), racial and ethnic minority (SVI 3), housing type and transportation (SVI 4), and whether or not they live in a rural area based on the patient zip code. Rural zip codes were identified using the Health Resources and Services Administration (HRSA) data files. The HRSA defines rural zip codes as those with fewer than 2,500 residents, in alignment with U.S. Census Bureau criteria [11]. Estimated household income (HHI) was defined as the average HHI of the patient’s zip code of residence.

Patient-reported outcomes during the preoperative and postoperative periods, at one month and three months postoperatively, were evaluated using the Quick Disabilities of the Arm, Shoulder, and Hand (DASH) instrument. QuickDASH is an 11-item questionnaire that assesses the physical function and symptoms of patients with upper extremity musculoskeletal disorders [12]. Higher scores indicate greater difficulty performing tasks or increased impairment of physical function [12].

Statistical analysis

Patients were grouped based on which surgery they had, CTR or TFR. Univariate analyses, including chi-square tests and two-sided independent sample t-tests, were used to determine demographic and QuickDASH differences between groups. Within each procedure, differences in QuickDASH scores at various time points were evaluated by demographics, geographic characteristics, and the social vulnerability index (SVI). Where variables were continuous, they were made categorical, with their median as the dividing point. Fisher’s exact test was performed when the assumptions of chi-square testing were not met. All statistical analyses were performed using R Studio (Version 1.4.1717© 2009-2022, RStudio, PBC). Statistical significance was assessed at p<0.05.

## Results

Of the 1,172 patients, 777 (66.3%) had a CTR, and 395 (33.7%) had a TFR. There were no differences in age, sex, race, marital status, rate of patients in rural areas, or household income (HHI) between procedure types. There were significant differences in social vulnerability in SVI 2 (household characteristics) (0.47 vs. 0.45; p=0.018) and SVI 3 (racial and ethnic minority composition) (0.39 vs. 0.36; p=0.043). Trigger finger patients had a higher average score when compared to carpal tunnel release patients. However, there was no significant difference in total SVI between the groups (Table 1).

**Table 1:** Patient demographics by procedure type p-values <0.05 in bold; data are expressed as mean ± SD or n (%); SVI: social vulnerability index

Demographics	All patients (n=1,172)	Carpal tunnel release (n=777)	Trigger finger release (n=395)	p-value
Age, years	61.53 ± 13.57	61.07 ± 13.79	62.44 ± 13.12	0.097
Female sex	699 (59.6)	477 (61.4)	222 (56.2)	0.099
Non-White race	209 (17.8)	131 (16.9)	78 (19.7)	0.254
Married/Life partner	701 (59.8)	461 (59.3)	240 (60.7)	0.683
Rural	53 (4.5)	36 (4.6)	17 (4.3)	0.914
Household income (in USD ($))	110,242 ± 23,741	110,236 ± 24,353	110,254 ± 22,520	0.991
SVI 1	0.34 ± 0.18	0.34 ± 0.18	0.34 ± 0.16	0.775
SVI 2	0.45 ± 0.15	0.45 ± 0.14	0.47 ± 0.15	0.018
SVI 3	0.37 ± 0.24	0.36 ± 0.24	0.39 ± 0.24	0.043
SVI 4	0.37 ± 0.20	0.37 ± 0.21	0.36 ± 0.20	0.477
Total SVI	0.33 ± 0.20	0.32 ± 0.20	0.33 ± 0.19	0.403

When comparing QuickDASH by procedure type, there was a significant difference in preoperative scores; CTR patients on average scored higher than TFR patients (46.4 vs. 39.8; p=0.004). Postoperatively, there was no difference at one or three months in QuickDASH scores between the two procedures (Table 2).

**Table 2:** QuickDASH by procedure type p-values <0.05 in bold; QD: QuickDASH

Time point	All patients		Carpal tunnel release		Trigger finger release		p-value
	N	QD Mean ± SD	N	QD Mean ± SD	N	QD Mean ± SD	
Preoperative	417	44.3 ± 22.1	280	46.4 ± 22.1	137	39.8 ± 21.3	0.004
1 month postoperatively	323	34.5 ± 21.5	212	34.5 ± 21.8	111	34.5 ± 21.1	0.995
3 months postoperatively	158	32.1 ± 18.9	115	33.0 ± 20.2	43	29.7 ± 15.0	0.268

When comparing QuickDASH scores by demographics for CTR patients, there was no difference in QuickDASH at any time point between those less than 65 years old and those 65 or older. Females, however, scored higher preoperatively (49.9 vs. 40.8; p=0.001) and at three months postoperatively (37.0 vs. 27.30; p=0.010) than males. White patients also scored higher preoperatively (56.4 vs. 44.4; p=0.003) and at one month postoperatively (45.6 vs. 32.7; p=0.016) than non-white carpal tunnel patients. Finally, those who were not married or did not have a life partner also scored higher preoperatively (51.7 vs. 43.0; p=0.002), one month postoperatively (39.7 vs. 31.6; p=0.015), and three months postoperatively (37.5 vs. 29.5; p=0.037) than those CTR patients who were married or had a life partner (Table 3).

**Table 3:** QuickDASH by demographics and procedure types P-values <0.05 in bold; Data are expressed as mean ± SD or n (%)

Carpal tunnel release												
QuickDASH	Age < 65 years	Age 65+ years	p-value	Male	Female	p-value	White	Non-White	p-value	Married/Life partner	Not married/Life partner	p-value
Preoperative	48.3 ± 22.4	44.0 ± 21.5	0.105	40.8 ± 20.9	49.9 ± 22.2	0.001	56.4 ± 25.0	44.4 ± 20.9	0.003	43.0 ± 20.5	51.7 ± 23.5	0.002
1 month postoperatively	37.2 ± 22.0	31.5 ± 21.3	0.06	31.7 ± 20.1	36.7 ± 22.9	0.098	45.6 ± 26.6	32.7 ± 20.5	0.016	31.6 ± 19.3	39.7 ± 25.0	0.015
3 months postoperatively	31.6 ± 19.2	35.1 ± 21.6	0.382	27.3 ± 19.2	37.0 ± 20.0	0.010	35.8 ± 19.9	32.1 ± 21.1	0.403	29.5 ± 19.4	37.5 ± 20.5	0.037
Trigger finger release												
QuickDASH	Age < 65 years	Age 65+ years	p-value	Male	Female	p-value	White	Non-White	p-value	Married/Life partner	Not married/Life partner	p-value
Preoperative	41.8 ± 19.8	37.6 ± 22.9	0.251	38.5 ± 20.1	40.9 ± 22.4	0.516	46.8 ± 21.3	38.1 ± 20.5	0.057	37.8 ± 20.8	43.3 ± 22.8	0.161
1 month postoperatively	36.7 ± 22.8	31.9 ± 18.8	0.229	28.9 ± 19.4	38.4 ± 21.5	0.018	45.5 ± 21.4	31.5 ± 20.1	0.007	35.2 ± 21.4	33.6 ± 20.9	0.696
3 months postoperatively	27.6 ± 14.1	32.4 ± 16.0	0.308	28.8 ± 13.5	30.1 ± 15.8	0.801	27.7 ± 14.5	30.4 ± 17.1	0.644	27.9 ± 15.8	32.1 ± 14.0	0.360

When comparing QuickDASH scores by demographics for TFR patients, there was no difference at any time point between those less than 65 years old and those 65 or older, or between those married or with a life partner and those who aren’t married or with a life partner. However, at one month postoperatively, females scored significantly higher (38.4 vs. 28.9; p=0.018) than male TFR patients, but there was no difference preoperatively or at three months postoperatively. Additionally, White patients scored higher at one month postoperatively (45.5 vs. 31.5; p=0.007) than non-White patients, but there was no difference preoperatively or at three months postoperatively for TFR (Table 3).

When comparing QuickDASH scores by geographic characteristics and procedure type, there were no significant differences in QuickDASH at any time point between rural and non-rural patients, patients above the median household income of $112,000, and those below the median household income, for both CTR and TFR patients (Table 4).

**Table 4:** QuickDASH by geographic characteristics and procedure types Data are expressed as mean ± SD; HHI: household income (in USD, $)

Carpal tunnel release						
Time point	Rural	Not rural	p-value	HHI < $112,000	HHI ≥ $112,000	p-value
Preoperative	39.9 ± 21.7	38.6 ± 16.5	0.837	48.9 ± 22.9	44.2 ± 21.2	0.079
1 month postoperatively	34.3 ± 21.3	41.9 ± 13.7	0.113	33.5 ± 21.3	35.3 ± 22.3	0.558
3 months postoperatively	37.8 ± 18.6	32.8 ± 9.7	0.154	32.4 ± 19.6	33.8 ± 20.7	0.713
Trigger finger release						
Time point	Rural	Not rural	p-value	HHI < $112,000	HHI ≥ $112,000	p-value
Preoperative	38.6 ± 21.7	39.9 ± 16.5	0.837	38.9 ± 21.5	40.8 ± 21.4	0.604
1 month postoperatively	41.9 ± 21.3	34.3 ± 13.7	0.351	31.2 ± 22.4	37.8 ± 19.4	0.097
3 months postoperatively	36.1 ± 0	29.6 ± 8.5	0.245	31.0 ± 17.6	28.8 ± 12.9	0.652

Finally, when comparing QuickDASH scores by Social Vulnerability Index (SVI) dimensions and procedure type, there were no significant differences in any dimension of social vulnerability for both CTR and TFR patients. The median for each individual dimension was used as the comparison point (Table 5).

**Table 5:** QuickDASH by Social Vulnerability Index Dimensions and procedure types All data presented as mean ± SD; SVI: Social Vulnerability Index

Carpal tunnel release															
Time point	SVI 1	SVI 1 ≥ 0.33	p-value	SVI 2	SVI 2 ≥ 0.47	p-value	SVI 3	SVI 3 ≥ 0.27	p-value	SVI 4	SVI 4 ≥ 0.30	p-value	Total	Total ≥ 0.30	p-value
Preoperative	45.0 ± 21.8	48.9 ± 22.9	0.149	47.2 ± 22.3	46.5 ± 22.7	0.776	44.9 ± 22.0	49.1 ± 22.7	0.137	46.4 ± 22.1	47.2 ± 22.7	0.783	45.5 ± 21.5	48.4 ± 23.4	0.294
1 month postoperatively	32.6 ± 21.2	37.5 ± 22.2	0.112	35.6 ± 21.9	33.4 ± 21.6	0.484	32.3 ± 20.8	37.5 ± 22.6	0.095	36.5 ± 21.3	32.9 ± 22.0	0.234	33.5 ± 21.6	36.0 ± 21.9	0.419
3 months postoperatively	32.7 ± 21.3	31.5 ± 18.8	0.771	34.2 ± 19.1	29.0 ± 21.0	0.188	31.4 ± 21.9	32.5 ± 18.8	0.798	32.9 ± 19.0	31.5 ± 20.7	0.706	33.7 ± 21.9	30.9 ± 18.4	0.474
Trigger finger release															
Time point	SVI 1	SVI 1 ≥ 0.33	p-value	SVI 2	SVI 2 ≥ 0.47	p-value	SVI 3	SVI 3 ≥ 0.27	p-value	SVI 4	SVI 4 ≥ 0.30	p-value	Total	Total ≥ 0.30	p-value
Preoperative	39.1 ± 22.0	40.7 ± 21.4	0.685	35.9 ± 23.3	43.5 ± 19.6	0.057	37.9 ± 20.9	41.7 ± 22.1	0.321	37.6 ± 24.3	42.0 ± 19.1	0.279	36.4 ± 22.7	43.0 ± 20.3	0.094
1 month postoperatively	36.4 ± 18.4	32.4 ± 22.8	0.322	34.5 ± 21.5	33.9 ± 20.9	0.883	33.1 ± 20.5	35.0 ± 21.6	0.637	30.5 ± 19.8	36.6 ± 21.6	0.140	32.3 ± 19.3	35.6 ± 22.3	0.412
3 months postoperatively	29.8 ± 13.7	29.3 ± 15.1	0.914	30.6 ± 15.4	28.5 ± 13.3	0.638	32.6 ± 16.0	27.9 ± 13.3	0.357	32.2 ± 14.8	26.9 ± 13.6	0.249	31.9 ± 14.8	27.5 ± 13.9	0.343

## Discussion

In the current study, patients undergoing CTR experienced greater levels of preoperative disability than those requiring TFR. Further, CTR patients resided in areas of greater social vulnerability related to household characteristics and racial or ethnic minority status. Postoperatively, sex and race were both associated with significant differences in levels of impairment in physical function for both procedures, with female and White patients demonstrating greater levels of upper extremity disability. In CTR patients, those who were not married reported higher levels of disability both preoperatively and postoperatively, suggesting that spousal support may play a key role in optimizing function. Notably, other socioeconomic factors, including rural geography, household income, and social vulnerability, were not associated with different levels of pre- or postoperative function for either patient population. While prior studies have described disparities in rates of surgical management of carpal tunnel and trigger finger conditions, to our knowledge, none have described disparities in the patient-reported outcomes of CTR and TFR procedures.

The majority of prior literature related to disparities in the treatment of carpal tunnel syndrome and trigger finger has focused on differences in rates of surgical treatment. When examining predictors for TFR surgery, Brodeur et al. found non-White race and increased social deprivation to be associated with decreased odds of surgery [13]. Further, women were more likely to undergo surgery after controlling for other factors. [13]. Social deprivation was also associated with lower odds of surgery for TFR [13]. Studies of CTS surgical treatment showed similar results with female patients; patients of non-White race and Hispanic ethnicity had decreased odds of undergoing surgery [14]. Further, increased social deprivation was also associated with lower odds of surgery for CTS [14]. All patients included in our study underwent surgical treatment; however, in comparison with the above studies, we found those who underwent TFR were associated with greater social vulnerability compared to CTR patients. While the single-institution nature of this study precludes our ability to draw broad conclusions regarding this finding, it highlights the need for future investigations regarding the cause of this trend.

While prior studies have evaluated demographic and socioeconomic disparities in the surgical treatment of CTR and TFR, other risk factors for complications, decreased function, and satisfaction have been described. Cognitive and mental health factors have been shown to influence surgical outcomes following CTR [15, 16]. A systematic review and meta-analysis on the effects of such interventions on the outcomes of CTR found a positive association between symptoms of depression and the severity of CTR symptoms following surgery [15]. Additionally, pain catastrophizing and depression were both associated with greater functional impairment following surgery in 100% and over 50% of the studies, respectively [15]. Depression was also associated with higher pain intensity following CTR [15]. Early return to work was associated with lower pain catastrophizing and anxiety [15]. Literature has also described a relationship between increasing depressive symptoms, pain catastrophizing, and decreased patient satisfaction following CTR [15-17]. While we did not evaluate the impact of preoperative mental health status on outcomes specifically, it is notable that decreased HHI or increased levels of social vulnerability were not associated with impaired outcomes in the current study. While prior studies have described higher rates of mental health disorders in socially and economically deprived areas, our results suggest that these geographic indicators may be of limited utility in the CTR and TFR populations [18]. Therefore, individualized assessment and management of a patient’s mental health may yield greater benefits to those undergoing CTR or TFR than broad, community-based interventions.

In the current study, our findings that lower household income, social vulnerability, and minority race were not associated with higher levels of upper extremity disability were unexpected. However, social and racial disparities have been well described in the literature among other orthopedic populations. In the spine surgery population, African American and Native American patients have been demonstrated to have a greater comorbidity burden and higher rates of complications than those of the White race [19]. In patients undergoing total joint arthroplasty, both race and socioeconomic status have been identified as risk factors for worse patient outcomes [20-23]. Stock et al. defined high-risk criteria as African American race, planned skilled nursing facility discharge, mental health or drug use issues, cardiac issues, or neurologic issues, and found those with one or more of these characteristics experienced longer lengths of stay and fewer home discharges [24]. Further, Weiner et al. found female patients, non-Hispanic Black patients, obese patients, Medicaid or uninsured status, patients older than 75, Charlson comorbidity index over three, and hip fracture diagnosis to be associated with an increased risk of an increased length of stay longer than two days as well as a non-home discharge [25]. In light of the findings of the current study and the trends observed across other musculoskeletal conditions, it appears that resources aimed at mitigating racial and socioeconomic disparities at the community level may be better allocated to alternative populations rather than those undergoing CTR or TFR. However, our finding that patients with less in-home support from a spouse or life partner experienced greater levels of postoperative disability suggests that an opportunity to provide these patients with additional support may exist. At our institution, the use of a novel outpatient home-based physical therapy program has been demonstrated to improve outcomes, including rates of successful home discharge, for total joint arthroplasty patients [26]. Therefore, we suggest similar programs, such as home-based occupational therapy, may provide hand surgery patients without in-home support with the assistance needed to maximize postoperative functional improvement.

This study does not come without limitations. First, as a single-institution study from a single geographic region, the population may not be representative of the broader population of TFR and CTR patients. Second, as an observational study, it is likely that selection bias exists in the populations that were deemed appropriate surgical candidates. Third, the follow-up window of our study was three months, which may have skewed the data for functional scores for those with delayed healing. Fourth, this study did not consider prior conservative treatment methods, which may confound preoperative QuickDASH scores. Finally, because not all patients completed outcomes surveys at each time point, we are limited in our ability to assess whether disparities in functional improvement, rather than absolute scores at each time point, exist.

## Conclusions

There is limited literature on investigating disparities in upper extremity surgery. However, our study found marital status, sex, and race to be associated with disparities in preoperative and postoperative physical function in patients undergoing carpal tunnel or trigger finger release. Therefore, future studies are warranted to verify these findings and develop solutions to disparities within the upper extremity specialty.
